# Characterization of chaotic dynamics in the human menstrual cycle

**DOI:** 10.1186/1753-4631-4-5

**Published:** 2010-10-05

**Authors:** GN Derry, PS Derry

**Affiliations:** 1 Department of Physics, Loyola University Maryland, Baltimore, MD 21210, USA; 2 Paula Derry Enterprises in Health Psychology, Baltimore, MD, USA

## Abstract

**Background:**

The human menstrual cycle is known to exhibit a significant amount of unexplained variability. This variation is typically dismissed as random fluctuations in an otherwise periodic and predictable system. Given the many delayed nonlinear feedbacks in the multiple levels of the reproductive endocrine system, however, the menstrual cycle can properly be construed as the output of a nonlinear dynamical system, and such a system has the possibility of being in a chaotic trajectory. We hypothesize that this is in fact the case and that it accounts for the observed variability.

**Results:**

Here, we test this hypothesis by performing time series analyses on data for 7749 menstrual cycles from 40 women in the 20-40 year age range, using the database maintained by the Tremin Research Program on Women's Health. Both raw menstrual cycle length data and a formal time series constructed from this data are utilized in these analyses. Employing phase space reconstruction techniques with a maximum embedding dimension of 12, we find appropriate scaling behavior in the correlation sums for these data, indicating low dimensional deterministic dynamics. A correlation dimension of D_c _≈ 5.2 is measured in the scaling regime. This result is confirmed by recalculation using the Takens estimator and by surrogate data tests. We interpret this result as an approximation to the fractal dimension of a strange attractor governing chaotic dynamics in the menstrual cycle. We also use the time series to calculate the correlation entropy (K_2 _≈ 0.008/τ) and the maximal Lyapunov exponent (λ ≈ 0.005/τ) for the system, where τ is the sampling time of the series.

**Conclusions:**

Taken collectively, these results constitute significant evidence that the menstrual cycle is the result of chaos in a nonlinear dynamical system. This view of the menstrual cycle has potential implications for clinical practice, modelling of the endocrine system, and the interpretation of the perimenopausal transition.

## Background

The prevailing biomedical view of the female reproductive system, exemplified by the menstrual cycle, has traditionally been that changes in various hormone levels cause further well-defined changes in a cyclically repeating pattern [1]. Despite this widespread view of menstruation, however, the empirical data show a high degree of variability that no current model accounts for [2]. Such variability is usually discounted as being due to random factors of no theoretical interest, but consideration of the dynamics inherent in this system suggests another explanation. The endocrine system governing the menstrual cycle has multiple nonlinear feedback loops involving at least six hormones produced by the ovaries, the pituitary gland, and the hypothalamus. This system can be modelled as a set of coupled nonlinear delay differential equations. Envisioning the menstrual cycle in this way as the output of a nonlinear dynamical system, chaotic solutions that would account for the observed variability are a distinct possibility. This paper presents the results of an experimental test of the hypothesis that the human menstrual cycle is in fact the output of such a chaotic regime in a nonlinear dynamical system, including a characterization of this regime by measurements of various relevant parameters using time series analysis.

The study of physiological systems using such techniques has been widespread, but includes only a small number of reports on endocrine physiology or the menstrual cycle. Prank et al. [3] analyzed the variation of parathyroid hormone levels in the blood over 24 hours (with a 2 minute sampling time) in three subjects, measuring the correlation dimension, Lyapunov exponents, and correlation entropy for these variations. Noguchi et al. [4] measured the blood levels of growth hormone and prolactin over 24 hours in six subjects (with a 30 minute sampling time), while Ilias et al. [5] measured blood levels of growth hormone and cortisol over 24 hours in ten subjects (again at 30 minute intervals). The latter two studies report values of attractor dimension estimates extracted from their data before and after sleep deprivation. All of this work, however, used relatively small numbers of time series data points, limited by the great difficulty of obtaining large amounts of physiological data for hormone concentrations in the blood and of obtaining such data over an extensive period of time. In this paper, we use a novel source of data to circumvent these problems. No studies, to our knowledge, have used nonlinear dynamical methods to study the time variation of female reproductive system hormones, but Bai et al. [6] did use such methods to examine the effects of ovarian hormones and thyroid-related hormones on heart rate variability during different parts of the menstrual cycle.

A number of mathematical models for the menstrual cycle have been developed. Bogumil et al. [7,8] developed an early but sophisticated model and simulation. Although nonlinearities are naturally built into this model, the simulation results required the addition of stochastic elements in order to exhibit the empirically observed variability in the menstrual cycle. This work was performed prior to a significant portion of our present physiological knowledge, however, and also prior to our present appreciation for the possibilities of chaotic dynamics in such systems. Increasingly accurate and sophisticated models have been developed and implemented more recently by Grigoliene and Svitra [9] and by Clark et al. [10] These models have been augmented by Reinecke and Deuflard [11] through the inclusion of more detail in the GnRH pulse generation, but this model has not been implemented yet. Although the recent models that have been implemented are able to reproduce measured hormone levels reasonably well over a single cycle, the observed variability of the cycles has not yet emerged naturally from these simulations. Other models have focused more on particular mechanisms such as the binding of hormones to receptors and calcium ion pumping through membranes [12,13]. The focus of attention has so far generally been on periodic solutions and predictability of the modelled variables. We hope to influence the course of future modelling investigations with the presentation of the results herein.

## Methods

**Figure 1:**
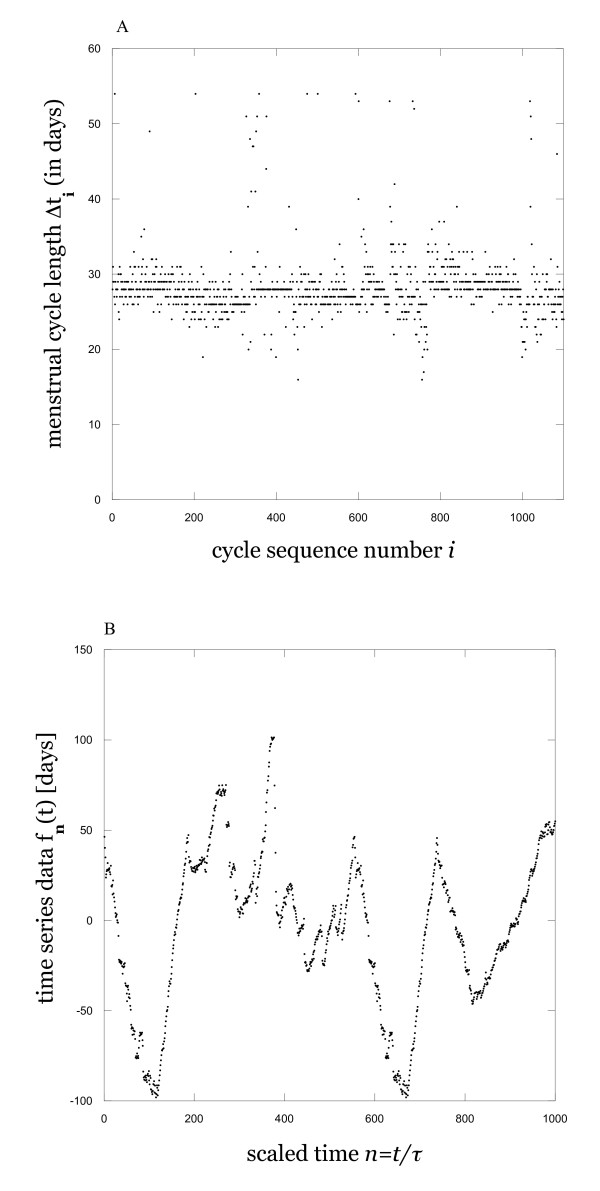
**Examples of data**. Menstrual cycle length data during 20-40 year age interval, illustrating typical observed variability (A), and time series constructed from the data using the protocol defined by Equation (1) in text (B). Illustrative subsets of total data record in both cases.

Data for the menstrual cycles used in this work was obtained from the database maintained by the Tremin Research Program on Women's Health [14], which contains the results of an ongoing longitudinal study begun in 1934 and includes data records for 3717 women. Since some of the subjects in the Tremin database have data records for more limited age ranges, we first isolated a subset of the subjects with longer records (minimally including the 20-40 year age range) and randomly selected those used in the present work from this set. Some of these subjects (< 10) were rejected due to documented health problems, missing data in the records, and so on. The analyses were ultimately performed on data for 20-40 years of age from 40 women, resulting in a total of 7749 menstrual cycle data points. In the Tremin research project, women prospectively record which days they are menstruating (and which not) on calendar cards, minimizing problems with inaccurate memory recall. The calendar card data was initially converted into a string of menstrual cycle times (defined as the time interval between the first day of menstruation for two consecutive menstrual events lasting at least two days). We only retained those menstrual cycles that were at least 16 days but no more than 54 days long; these were the 5^th ^and 95^th ^percentiles for menstrual cycle length reported for the Tremin population [2] (this protocol eliminated pregnancies, undocumented health problems, and so on). Also, in keeping with definitions used by the Tremin researchers, there had to be at least a two-day gap between bleeding episodes to count as a new cycle. The data for all women is then concatenated. A subset of the resulting menstrual data sequence is shown in Figure 1A.

The data was also converted into a formal time series by defining a sampling time τ such that t_n _= nτ. The n^th ^term of the time series f(t) is then defined as

(1) $$f_{n}\equiv \left(\sum_{i=1}^{n}\Delta t_{i}\right)-n\tau$$

where Δt_i _is the i^th ^menstrual cycle time length in the sequence. Equation (1) is essentially a device by which to create a formal time series using the information content available in the menstrual cycle time data. The rationale that underlies the validity of this device is based on the fact that the onset of menstruation is a discrete event that occurs when the body's endocrine system achieves some specific state, and that this state is by hypothesis deterministically related to all preceding and following states, since we are assuming that the endocrine system is indeed a dynamical system. It then follows that the time separating this state from the state of the system at time t_n _is likewise a function f(t) of the state of the system, and it is this function that we have sampled the n^th ^measurement of with our definition. Although this is an unconventional sort of time series, being a time difference itself rather than some other separately measured variable, the reasoning that underlies it is basically the same as that which underlies the validity of the much-used phase space reconstruction techniques [15]. For each individual woman, the sampling time was set equal to the average cycle length to eliminate stationarity. The final step in this construction is the concatenation of the data together into the time series used for analysis. A similar concatenation procedure has also been used in previous physiological studies [5]. An example of the resulting time series for a subset of the data is shown in figure 1B.

We perform analyses using both this formal time series that we constructed and also the raw inter-event time data, Δt_i_, treated as a series. The work of Castro and Sauer [16] has shown that such inter-event time data can be used to calculate correlation dimensions for two different models of event generation from the dynamical behavior of a system. The disadvantage of this method is that we don't know the details of the event generation mechanism for our present experimental case of a real physiological system. The disadvantages of the formal time series are that it may artificially introduce autocorrelation into the series (which, however, can be corrected for) and the justification for it is not truly rigorous. We argue, however, that concordance between the results of the analysis using the Δt_i _and the results using the *f_n _*strongly suggests that both are valid.

A number of analyses were performed on this menstrual cycle data. Our objective, in part, was to look for evidence of chaos governing the dynamics of this system by examining the data for signs of a strange attractor. This was done using standard techniques, namely by performing a state space reconstruction with time delay embeddings [15] for the time series with time delay ΔT = 7τ for all of the analyses reported here, and simply using ΔT = τ for the cycle time sequence as outlined in Castro and Sauer [16].

For the time series, the correlation dimension D_c _of the time series was calculated using the Grassberger-Procaccia method [17]. Correlation sums C(R) were calculated for R values from R = 2 to R = 125. The correlation dimension

(2) $$D_{c}=\underset{R\to 0}{\lim}\underset{N\to \infty }{\lim}\frac{d[\log C(R)]}{d[\log R]}$$

can then be found from the slope of a log[C(R)] *versus *log[R] plot over some appropriate scaling region, and the D_c _obtained by this method is known to be a good approximation to the fractal dimension of a strange attractor that has generated the time series data. This procedure was repeated for embedding dimensions ranging from d = 1 to d = 12. Since a strange attractor only fills a limited volume of the available state space, values of D_c _that remain roughly constant as d increases indicate the existence of a low dimensional attractor governing the dynamics, and hence the likelihood of a deterministic chaotic system. Since data points closer in time are forced to be near each other by the time series construction protocol, we did not use the first 70 data points (nearest the point in question for that term of the summation) in the correlation sums, employing a correction for this autocorrelation problem suggested by Theiler [18]. For the inter-event timing data (i.e. menstrual cycle lengths), a similar procedure is used, in this case constructing the embedding vectors out of consecutive values in the sequence. Correlation sums are computed in the same way and values of D_c _found from the slopes of log[C(R)] *versus *Log[R] plots. Because the amplitude of this sequence is smaller than that of the time series, the scaling range of R is also considerably smaller, but the scaling is quite good within that range. Autocorrelation is minimal for this sequence, so no Theiler correction is needed (this fact was verified empirically).

## Results

**Figure 2:**
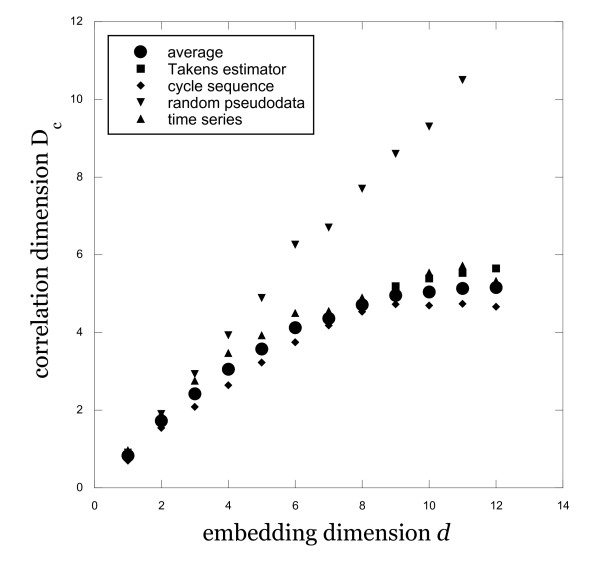
**Correlation dimension results**. Variation of the correlation dimension with increasing embedding dimension, using all available data and a variety of computational methods. Results for random pseudodata are also shown for comparison.

Results for both the time series and for the inter-event time sequence are shown in figure 2. Also shown in Figure 2 are results for the correlation dimension at high values of d calculated using a different method, namely the Takens estimator [19]. Using the time series data for these computations, the Takens estimator varied with R but remained stable for a range of R values in the scaling region. The results in Figure 2 are those in the middle of this range, for R ≈ 30, with a Theiler correction of 70 as in the previous analysis. The scatter in these results offers a sense of the uncertainty in the correlation dimension measurement and of its reproducibility using differing methodologies. Average values for the correlation dimension, using all of these results, are plotted in Figure 2 as well. Considering the scatter for the various computational methods, our best value for the correlation dimension is D_c _= 5.2 ± 0.7 in this system. For comparison, the correlation dimension for a set of Gaussian random numbers with a mean and range similar to the menstrual cycle data is also plotted in Figure 2. These computations result in D_c _≈ d as expected, in contrast to the computations using the real data.

To test for potential artifacts due to non-randomness in the data from correlated noise (which can masquerade as deterministic chaos by yielding low correlation dimensions), we recalculated D_c _using surrogate data sets generated from the inter-event time sequence [20]. The surrogate data was generated by randomizing the phases of the Fourier transform of the real data and then inverse transforming the resulting series. For the real data, the Takens estimator for d = 10 and R = 5.5 is computed to be ≈ 4.5, whereas for the surrogate data the Takens estimator is computed to ≈ 8.8. This procedure was repeated using two 2500 point subsets of the data, with similar results. Hence, the surrogate data tests indicate that the low correlation dimensions are the result of deterministic chaotic dynamics, not artifacts.

**Figure 3:**
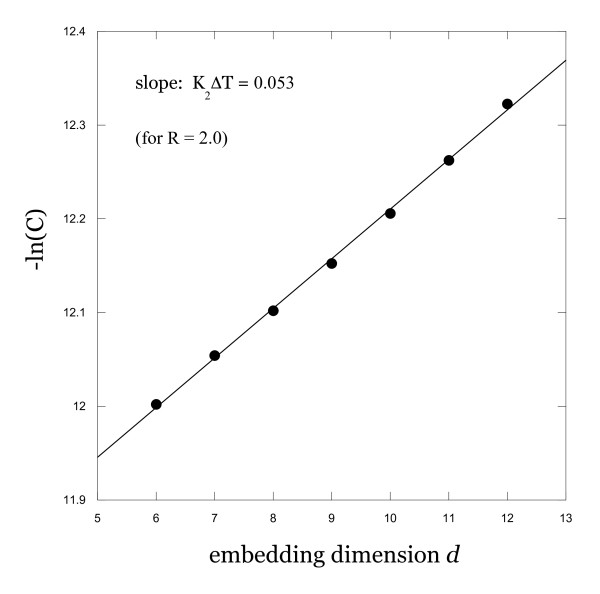
**Correlation entropy results**. Logarithmic variation of the correlation sums with embedding dimension for a fixed R in the low R limit, illustrating the expected behavior for a chaotic system. Slope of the resulting straight line yields the correlation entropy.

We can also obtain dynamical information about the system, in addition to the more geometric characterization of its attractor that we have found, from the correlation sums. These correlation sums can be employed [21] to compute the correlation entropy,

(3) $$K_{2}=\frac{1}{\Delta T}\ln\left[\frac{C(d,R)}{C(d+1,R)}\right],$$

which approximates the Kolmogorov-Sinai entropy of a chaotic system. From this relationship, we see that a plot of -ln[C(d)] *vs *d for a fixed R should be a straight line with slope K_2_ΔT. For the smallest experimentally accessible value of R and for large d (consistent with the theoretical validity of Equation 3 in the R→0 and d→∞ limits), we observe behavior consistent with this predicted relationship, as shown in Figure 3. From the slope of the resulting line, we find that K_2 _≈ 0.008/τ for this system, where τ is the sampling time used to construct the time series. The fact that the K_2 _value computed in this way has the correct sign and that the overall behavior of K_2 _with variations in d and R is consistent with our expectations for chaotic systems offers further evidence of non-linear dynamics in the menstrual cycle.

**Figure 4:**
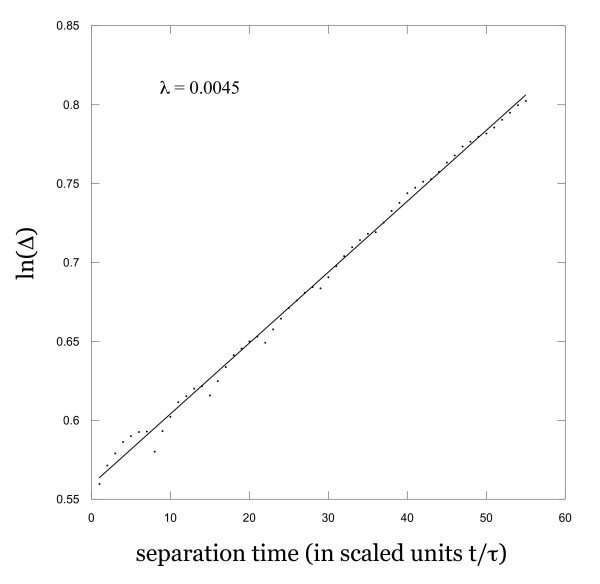
**Logarithmic trajectory divergence**. Logarithmic separation of trajectories for initially nearby points; slope is theoretically equal to the maximal Lyapunov exponent.

Another important characterization of the dynamics and topology of a system is its spectrum of Lyapunov exponents. At least one positive exponent is a necessary and sufficient condition for the system to be chaotic. The data here do not have enough resolution to compute the full spectrum of Lyapunov exponents, but we are able to compute the largest exponent using a robust and straightforward method suggested by Rosenstein et al. [22] and by Kantz [23]. Basically, we find all of the points in an embedding space of the time series that are close a given point (i.e. within some specified distance ε at widely separated times), and compute the separation Δ between each of these points and the given point for a moderate sequence of time steps (we used 55). The value of ln(Δ) is found for each of these time steps and averaged over all of the points at each time. This procedure is repeated for a large number of sufficiently time-separated given initial points until virtually all parts of the attractor have been effectively sampled, and the resulting ln(Δ) values are again averaged. When the average ln(Δ) values are plotted versus time, a straight line should result for a chaotic system, and the slope of this line is the value of the largest Lyapunov exponent. Application of this method to the time series constructed from the menstrual cycle data yields the results shown in Figure 4. This result is indeed observed to be approximately linear, with a positive slope of 0.0045, yielding a largest Lyapunov exponent of λ ≈ 0.005/τ. The procedure was repeated for several values of ε and d, with all results consistent and reproducible. We interpret these results to be another signature of chaotic dynamics in the menstrual cycle. In addition, the values of K_2 _and λ are entirely consistent with Pesin's Identity, further corroborating our conclusions.

## Discussion

One of the interesting features of the results reported here is the comparatively low dimensionality of the system. The phenomenological details of the human menstrual cycle are extraordinarily complicated. Reinecke and Deuflhard [11], for example, have devised a model for the human menstrual cycle consisting of 43 differential equations with 191 parameters, and yet an attractor with a fractal dimension of ≈5.2 characterizing the dynamics of the human menstrual cycle suggests that only 6 degrees of freedom may be needed to describe these dynamics. This will hopefully stimulate a rich array of novel approaches to identify the agents that appear to govern the dynamics of the menstrual cycle, and it offers the possibility that simpler systems of equations may then be possible to analyze these dynamics.

The second important implication for modeling results is that the variability of the menstrual cycle, which has been well documented for many years, is a natural feature of the dynamics itself. This variability is not merely due to random fluctuations or external interference, and thus any results generated by implementations of model systems that do not exhibit this variability imply that the models have not captured some important part of the system's dynamics. Variability in the menstrual cycle should not be ignored by only considering average behaviors as is typical, nor imposed by means of *ad hoc *stochastic additions as Bogumil et al. [7,8] did. Instead, the variability is intrinsic to the behavior of the chaotic system and valid models should reproduce comparable variability as a natural outcome of their implementations. Moreover, such models can in principle be tested, and parameters optimized, by comparing the various nonlinear measures reported in the present paper (D_c_, K_2_, and λ) to the output of the models. We would assert that any model producing only perfectly periodic menstrual cycles is, at best, incomplete.

Beyond the details of particular models, the discovery of chaotic dynamics in the menstrual cycle has implications for the more general paradigmatic approach that is taken with regard to its behavior and the interpretation thereof. For example, an increase in variability might be construed as a natural consequence of the dynamics of the system rather than as a pathological deviation from normal behavior. Clinical goals associated with control and predictability may not be appropriate for a system that is known to be chaotic, and pharmacological interventions that impose regularity might need to be re-examined in light of this new context. The prevailing view of menopause as a senescent breakdown of the system should also be reconsidered in light of the idea that the menstrual cycle is the output of a nonlinear dynamical system and therefore might have a variety of possible regimes characterized by different values of relevant control parameters. Reinterpretations of this sort will be considered in more detail elsewhere, but far more analytical work is needed before any definitive conclusions are possible.

We are particularly interested in the issue of menopause and the perimenopausal transition. We are analyzing menstrual cycle data for the perimenopause in order to characterize the chaotic dynamics in that regime and compare it to the results of data from 20-40 year old women presented in this report. Significantly different values of the relevant measures (such as D_c_, K_2_, and λ) during the perimenopause would indicate that the dynamical system (i.e. reproductive endocrine physiology) had undergone a phase transition to some new attractor, a conclusion that seems more consistent with existing evidence than the usual notions of senescence, breakdown, and pathology. However, it is likely that refinements in the analysis to improve the precision of these measurements are needed in order to enact this agenda.

## Conclusions

The major conclusion of the present paper is that the human menstrual cycle is in fact the output of a nonlinear dynamical system in a chaotic regime, even in the most comparatively regular phase of its development during the 20-40 year age range. A quantitative characterization of this trajectory is provided by its correlation dimension D_c _= 5.2 ± 0.7, correlation entropy K_2 _≈ 0.008/τ, and largest positive Lyapunov exponent λ ≈ 0.005/τ (where τ represents the sampling time of the data). We believe that the evidence presented here for the chaotic nature of the menstrual cycle is persuasive and that our quantitative measures of its dynamics are reliable.

## Competing interests

The authors declare that they have no competing interests.

## Authors' contributions

GD developed and implemented the mathematical algorithms used in the analysis and wrote most of the manuscript draft. PD had the initial idea for the study and contributed most of the ideas concerning the implications for how menstruation is conceptualized. Both authors contributed to the development of the data set, and both authors read and approved the final manuscript.
